# Significant hindlimb static weight-bearing asymmetry persists for 40-weeks in a longitudinal study in two widely used rat models of surgically induced osteoarthritis knee pain

**DOI:** 10.3389/fphar.2025.1560265

**Published:** 2025-04-16

**Authors:** A. Kuo, A. Raboczyj, J. R. Nicholson, L. Corradini, M. T. Smith

**Affiliations:** ^1^ School of Biomedical Sciences, Faculty of Health, Medicine and Behavioural Sciences, The University of Queensland, St Lucia, QLD, Australia; ^2^ Boehringer Ingelheim Pharma GmbH & Co. KG, Biberach an der Riss, Germany

**Keywords:** osteoarthritis knee pain, static weight bearing asymmetry, pain behavior in rat, anterior cruciate ligament transection (ACLT), ACLT + medial meniscectomy (MMx)

## Abstract

**Introduction:**

Unrelenting osteoarthritis (OA) knee pain is the primary reason patients seek treatment that may ultimately result in knee replacement surgery. Although the anterior cruciate ligament transection (ACLT) and the ACLT plus medial meniscectomy (MMx) induced rat models of OA knee pain are well-characterized histologically, reports on changes in pain-like behaviors that persist longterm, are scant and so this is a knowledge gap.

**Methods:**

We conducted a 40-week longitudinal study using these models in male Sprague-Dawley rats. Hindlimb static weight-bearing asymmetry was assessed using the incapacitance test. Von Frey filaments and an Analgesy-Meter were used to measure paw withdrawal thresholds (PWTs) and paw pressure thresholds (PPTs) respectively in the hindpaws.

**Results and discussion:**

Our findings show significant, reproducible and long-lasting static weight-bearing asymmetry in the hindlimbs of both models (but not the sham-control group) for the 40-week study duration. Significant mechanical hypersensitivity developed in the ipsilateral hindpaws of the ACLT + MMx model (PWTs ≤8 g) which reversed spontaneously by ∼8–12-weeks. In the ACLT and the sham-groups, significant mechanical hypersensitivity did not develop in the ipsilateral hindpaws. In conclusion, hindlimb static weight-bearing asymmetry is a long-lasting, significant pain behavioral endpoint in these models suitable for assessing novel disease-modifying OA therapeutics and/or analgesic drug candidates aimed at alleviating unrelenting chronic OA knee pain in patients.

## 1 Introduction

Reduced function and quality of life is a major reason why patients with painful knee osteoarthritis (OA) seek medical treatment that may result ultimately in knee replacement surgery (Malfait and Schnitzer, 2013; Price et al., 2018). Although non-steroidal anti-inflammatory drugs are the most effective oral medications for the relief of OA knee pain, they are associated with important adverse effects. These include gastrointestinal (GI) bleeding as they block the enzyme, cyclooxygenase 1 (COX-1) (Chou et al., 2011). In people taking NSAIDs, the 1-year risk of serious GI bleeding ranges from 1 in 2,100 in adults under the age of 45 years to 1 in 110 for adults over the age of 75 years (Chou et al., 2011). For people taking selective COX-2 inhibitors such as celecoxib, they are associated with an increased risk of serious cardiovascular harms (Chou et al., 2011). Hence, there is a large unmet medical need for a new generation of therapeutics for improving the relief of OA knee pain.

The most prominent histologic characteristic of knee OA is articular cartilage erosion (Zhu et al., 2020). The homeostasis and integrity of articular cartilage depends upon the biomechanical and biochemical interplay with subchondral bone and other joint tissues (Zhu et al., 2020). However, reliance solely on histologic methods to characterize rodent OA models is at odds with radiographic imaging of OA in humans where changes seen do not always correlate with pain severity reported by patients (Jacobs et al., 2014).

To investigate the pathophysiology of OA knee pain, rodent models of surgically induced knee OA were developed including the anterior cruciate ligament transection (ACLT) model (Stoop et al., 2001), the destabilization of the medial meniscus model (Glasson et al., 2005), medial meniscus transection (Allen et al., 2012) and the ACLT plus medial meniscectomy (MMx) model (Roberts et al., 2003). These models have translation value for identifying novel targets for discovery of new disease-modifying OA therapeutics and/or analgesic agents for improved relief of OA knee pain. Availability of novel, highly efficacious, well-tolerated analgesics for relieving OA knee pain have the potential to delay, and/or reduce the need for knee replacement surgery.

In previous work by others using the ACLT and the ACLT + MMx rat models of knee OA in a 10-week study design, histologic assessment showed articular cartilage damage within 2-weeks with detectable cartilage surface damage and proteoglycan loss as early as 1-week post-surgery (Hayami et al., 2006). Subchondral bone loss in the knees of both models was evident within 2-weeks and this was followed by significant increases in subchondral bone volume relative to sham-controls, that persisted for the 10-week study period (Hayami et al., 2006). The incidence of osteophyte formation was highest at 10-weeks in the ACLT model and at 6-weeks in the ACLT + MMx model (Hayami et al., 2006). In a 20-week longitudinal study (Appleton et al., 2007) in the ACLT + partial MMx (pMMx) rat model, cartilage degradation, histologic subchondral changes and subchondral bone loss were present by 2-weeks after surgery to induce this model. There was also increased chondrocyte hypertrophy and forced mobilization exercise accelerated OA progression in the destabilized joint (Appleton et al., 2007). However, in these studies, pain-like behaviors was not assessed, even though pain is the highest unmet medical need for patients with OA knee pain (Jacobs et al., 2014). In previous work by others, pain behaviors including dynamic weight bearing, incapacitance (static and during jump), mechanical allodynia and hyperalgesia and gait analysis were assessed, but most of these studies were short-term in nature with conflicting results in some instances.

To address this issue, here we report the outcomes of a 40-week longitudinal study to assess hindlimb static weight-bearing asymmetry as a reproducible and long-lasting pain behavioral endpoint relative to knee edema and mechanical hypersensitivity in the hindpaws, in both the ACLT and the ACLT + MMx rat models of OA knee pain.

## 2 Materials and methods

### 2.1 Drugs, chemicals and reagents

Isoflurane was from Provet Australia (Northgate, Brisbane, Australia) and medical oxygen was from Coregas Pty Ltd. (Darra, Brisbane, Australia). Sodium benzylpenicillin (Benpen™) vials were from CSL Limited (Sydney, New South Wales, Australia). Sterile water for injection (BP) vials were from Pfizer (Sydney, New South Wales, Australia).

### 2.2 Animals and housing

**FIGURE 1:**
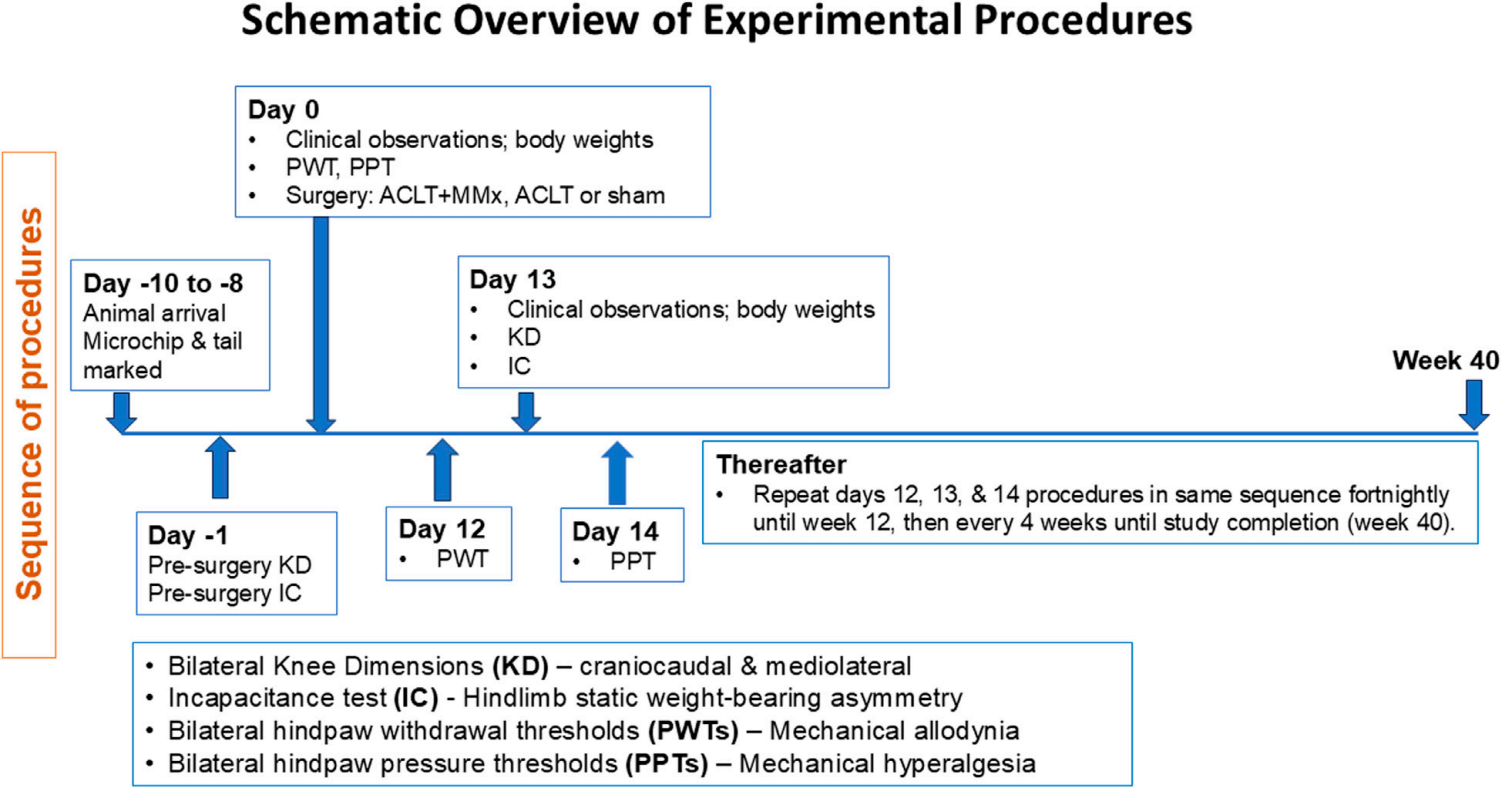
Schematic overview of the experimental procedures and pain behavioral assessments performed during this 40-longitudinal study in two surgically induced rat models of unilateral knee osteoarthritis (ACLT + MMx and ACLT) relative to a sham-operated group.

Ethics approval was from the University of Queensland Animal Ethics Committee (Approval Numbers CIPDD/TETRAQ/446/12/ARC and CIPDD/418/15/ARC) and this study was conducted in accordance with the requirements of the Australian Code of Practice for the Care and Use of Animals for Scientific Purposes (eighth edition, 2013). Male Sprague-Dawley (SD) rats were from the Animal Resources Centre (Perth, WA, Australia) and they were used to surgically induce OA of the left knee (ACLT and ACLT + MMx models) or perform a sham procedure. Rats were housed in groups of up to three in individually ventilated cages containing recycled paper bedding (Fibrecycle Pty Ltd., Yatala, Qld, Australia) in a holding facility with a 12 h/12 h light–dark cycle. Rats had free access to water and rodent chow and environmental enrichment comprised red rodent hutches, Kimwipes and rat Chewsticks in each cage. Rats were given at least 4 days of acclimatization to the animal holding facility prior to initiation of any procedures. The experiments were performed in the light phase and a schematic overview of the experimental procedures is shown in Figure 1.

### 2.3 General health

Body weights and clinical observations of individual rats were recorded pre-surgery (week 0) and at regular intervals post-surgery (2, 4, 6, 8, 10, 12, 16, 20, 24, 28, 32, 36 and 40-weeks) for the 40-week longitudinal study duration as an index of general health.

### 2.4 Surgical induction of OA knee pain in rats

This was an exploratory study involving two widely utilized surgically induced rat models of OA knee pain (viz the ACLT and ACLT + MMx models) and a group of rats that underwent sham surgery. A sample size of n = 8 per group was used. Sense checking showed that this sample size would provide 80% power with an alpha value of 0.05 to detect a difference between the groups of 33% with 25% inter-animal variability in the behavioral readouts. A between-group difference in pain behavioral endpoints of at least 30% is required for pharmacological intervention studies.

#### 2.4.1 ACLT model

Rats (∼200 g; n = 8) were anaesthetized using 3% isoflurane delivered in oxygen and a subcutaneous injection of benzylpenicillin (60 mg) was given. The left knee was shaved and the skin was cleaned with 70% ethanol and betadine. As described by others (Hayami et al., 2006), an incision (∼1 cm) was made across the left knee, blunt dissection was performed around the joint and a parapatellar muscle incision (∼1 cm) was made from the medial aspect of the joint. With the knee in full flexion, the patella was displaced laterally and the articular cartilage and femur were exposed. After surgical transection of the anterior cruciate ligament with micro-scissors, the joint was flushed with sterile saline and sterile vicryl 4-0 braided absorbable sutures were used to close the incision in layers and topical local anesthetic was applied to the sutured wound. Animals were kept warm and monitored closely during post-surgical recovery before being returned to the animal holding facility and housed in pairs.

#### 2.4.2 ACLT + MMx model

Rats (∼200 g; n = 8) underwent a similar procedure to that described above for the ACLT model. Specifically, rats were anaesthetized using 3% isoflurane delivered in oxygen and a subcutaneous injection of benzylpenicillin (60 mg) was given. The left knee was shaved and the skin was cleaned with 70% ethanol and betadine. An incision (∼1 cm) was made across the left knee, blunt dissection around the joint was performed and a parapatellar muscle incision (∼1 cm) was made from the medial aspect of the joint. With the knee in full flexion, the patella was displaced laterally and the articular cartilage and femur were exposed. Micro-scissors were used to transect the anterior cruciate ligament, the medial collateral ligament, and the medial meniscus was resected (Hayami et al., 2006). After completion of these procedures, the joint was flushed with sterile saline and the incision was closed in layers and topical local anesthetic was applied to the sutured wound. Animals were kept warm and monitored closely during post-surgical recovery before being returned to the rodent holding facility and housed in pairs.

#### 2.4.3 Sham-control group

For sham-operated rats (∼200 g; n = 8), a similar procedure was performed on the left knee of rats anaesthetized with 3% isoflurane delivered in oxygen to that described in the preceding sections including subluxation of the patella, but without transection of either the anterior cruciate ligament or the medial collateral ligament and the medial meniscus was not resected. The joint surface was flushed with sterile saline and the incision was closed using sterile sutures (Hayami et al., 2006) and topical local anesthetic was applied to the sutured wound. Animals were kept warm and monitored during post-surgical recovery before being returned to the animal holding facility and housed in pairs.

### 2.5 Pain behavioral assessments

Pain behavioral testing was conducted in a purpose-built pain behavioral facility during the light phase with piped soft music as white noise. Each pain behavioral test was conducted by a tester blinded to treatment group at regular intervals in a dedicated room at ambient temperature (21 ± 2°C; mean ± SD).

#### 2.5.1 Knee edema

A vernier caliper was used to measure the craniocaudal and mediolateral dimensions of both the left (ipsilateral) and the right (contralateral) knees for each rat in each of the ACLT + MMx, ACLT and sham-groups (n = 8/group) in this study prior to surgery and at the following post-surgical times (2, 4, 6, 8, 10, 12, 16, 20, 24, 28, 32, 36, 40 weeks). Measurements were performed by testers blinded to the treatment group.

#### 2.5.2 Pain behavioral testing

##### 2.5.2.1 Incapacitance test of hindlimb static weight bearing asymmetry

In week −1, all rats underwent training and acclimatization to the incapacitance test device (Columbus Instruments, Columbus, Ohio, United States). On the day of testing, animals were acclimatized in their home cages to the room where the incapacitance testing device was located for approximately 10-min prior to test initiation. Next, individual rats from the ACLT, ACLT + MMx and sham-groups (n = 8/group) were acclimatized to the plexiglass restrainer of the incapacitance test device, standing with the hindpaws resting comfortably on two separate sensor force plates and facing forward (15–30 min) as described by others (Bove et al., 2003). The ratio of weight distribution between the contralateral (non-injured side) and the ipsilateral (injured side) hindlimbs was measured over 2–3 s. The downward force applied on each sensor was displayed and recorded and 3 replicate readings were obtained. If during the 2–3 s period for incapacitance data capture, animals attempted to turn around or get out of the device, the reading was discarded and then repeated. Static weight-bearing was assessed pre-surgery and at 2, 4, 6, 8, 10, 12, 16, 20, 24, 28, 32, 36 and 40-weeks post-surgery; n = 8/group). Hindlimb static weight-bearing measurements were performed by testers blinded to the treatment group.

##### 2.5.2.2 Assessment of mechanical allodynia in the hindpaws

On each testing occasion for the ACLT, ACLT + MMx and sham-groups, rats were acclimatized individually to wire mesh testing cages for 15–30 min prior to assessment of paw withdrawal thresholds (PWTs) for each of the hindpaws using calibrated Semmes-Weinstein von Frey filaments (Stoelting Co., Wood Dale, IL, United States) (Muralidharan et al., 2016). Commencing on day −1 or day 0 and continuing until study completion at week 40 post-surgery, filaments that delivered forces of 2, 4, 6, 8, 10, 12, 14, 16, 18 and 20 g were used to apply graded non-noxious pressure to the plantar surface of the footpad of each hindpaw for each rat using the up-down method we have described previously (Shenoy et al., 2017). On each testing occasion, three measurements for each hindpaw were recorded with successive readings separated by ∼5-min intervals. The mean ipsilateral and contralateral PWTs were calculated for each hindpaw for each testing time-point. Mechanical allodynia was regarded as fully developed in individual rats when the mean ipsilateral hindpaw PWT was ≤8 g (∼30% decrease from the pre-surgery baseline PWT). PWT measurements were performed by testers blinded to the treatment group.

##### 2.5.2.3 Assessment of mechanical hyperalgesia in the hindpaws

Rats from each of the ACLT + MMx, ACLT and sham-groups were gently restrained in a light towel and an Analgesy-meter (Ugo Basile, Italy) was used to apply graded noxious pressure to the hindpaws to determine the paw pressure thresholds (PPTs) (Shenoy et al., 2017). In brief, each hindpaw was placed individually on a small plinth beneath a cone-shaped pusher with a rounded tip to avoid tissue damage. The pusher was used to apply a noxious pressure stimulus of increasing force to the hindpaw and the threshold at which the rat exhibited a withdrawal response, was recorded. A maximum cut-off force of 200 g was used to prevent hindpaw injury. On each testing occasion, three measurements for each hindpaw were recorded with successive readings separated by ∼5-min intervals. The mean PPTs for each hindpaw on each testing occasion (pre-surgery and at 2, 4, 6, 8, 10, 12, 16, 20, 24, 28, 32, 36 and 40-weeks post-surgery) were calculated and recorded. Mechanical hyperalgesia was classified as fully developed when the mean ipsilateral hindpaw PPT values for individual animals were ≤90 g (∼30% decrease from the pre-surgery baseline PPT). PPT assessments were performed by testers blinded to the treatment group.

### 2.6 Study completion

After pain behavioral measures were assessed at 40-weeks of the study period, animals were euthanized on schedule with an overdose of pentobarbital.

### 2.7 Data and statistical analysis

Two-way ANOVA with Tukey’s multiple comparison test was used to compare the body weights between groups for the 40-week study period. One-way ANOVA with Dunnett’s multiple comparisons test was used to compare each of the craniocaudal and mediolateral Δ knee diameter AUC values for each of the ACLT + MMx and ACLT groups relative to the sham group. One-way ANOVA with Tukey’s multiple comparison test was used to compare the ΔPPT AUC values and the ΔPWT AUC values between the ACLT, the ACLT + MMx and the sham-groups. One-way ANOVA with Tukey’s multiple comparison test was also used to compare the body weight normalized ΔIncap AUC values between the groups. For one-way ANOVA, the F values together with their associated degrees of freedom (treatment, interaction/residual) are reported. For two-way ANOVA, F values together with their associated degrees of freedom (treatment, time, interaction/residual) are reported. For the Dunnett’s and Tukey’s multiple comparisons tests, the multiplicity adjusted P values were calculated, and these are reported in the Results section and are shown on the relevant figure panels. GraphPad™ Prism (version 10.1.2) was used for statistical analyses and the statistical significance criterion was P < 0.05.

## 3 Results

### 3.1 General health

**FIGURE 2:**
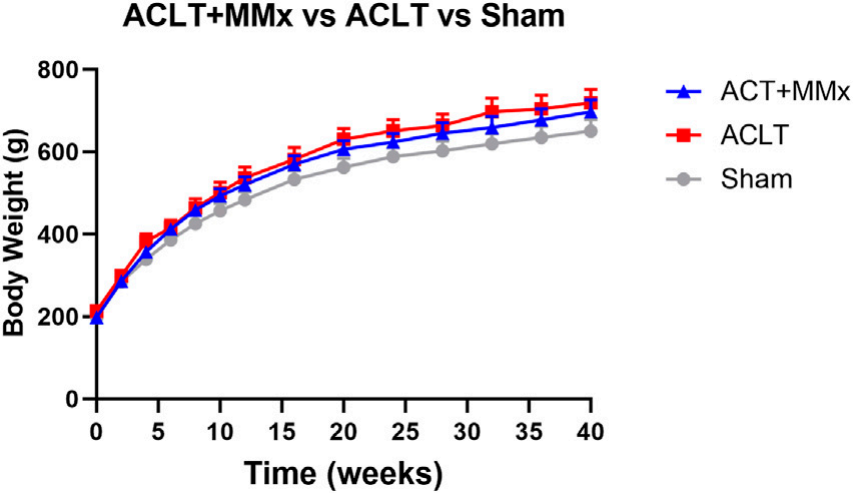
Mean (±) SEM body weights of rats increased in a temporal manner over the 40-week study period indicative of good general health. Although the mean (±) SEM body weights of the ACLT + MMx and ACLT groups of rats (n = 8/group) did not differ significantly, they were significantly different from that of the sham group (n = 8), albeit <10% heavier. Two-way ANOVA (F_2, 294_ = 18.86, P < 0.0001) with Tukey’s multiplicity adjusted P values of P > 0.05, P < 0.001 and P < 0.0001 for comparisons between the ACLT + MMx and ACLT groups, the ACLT + MMx and the sham group, and the ACLT and sham-groups respectively.

The mean (±SEM) pre-surgery body weights were 198.3 (±3.1), 213.3 (±4.7) and 198.0 (±2.1) g for the ACLT + MMx, ACLT and sham group respectively and these did not differ significantly (P > 0.05) between the 3 groups. For rats in each of these groups, mean (±SEM) body weights increased in a temporal manner over the 40-week study period (Figure 2), showing that the animals had good general health for the study duration. The mean (±SEM) body weights differed between the 3 groups (F_2, 294_ = 18.86, P < 0.0001) over the 40-week study period. Further analysis using Tukey’s multiple comparisons test showed that the mean (±SEM) body weights for the ACLT + MMx and ACLT groups did not differ significantly from each other, but the body weights of both surgical groups differed significantly from that of the sham group with the corresponding multiplicity adjusted P values being P > 0.05, P < 0.001 and P < 0.0001 respectively. It should be noted that the heavier mean (±SEM) body weights of the ACLT + MMx and ACLT groups relative to the sham-group were of small magnitude at <10% (Figure 2). There were no overt adverse signs observed in rats during this 40-week longitudinal study.

### 3.2 Change in knee dimensions

**FIGURE 3:**
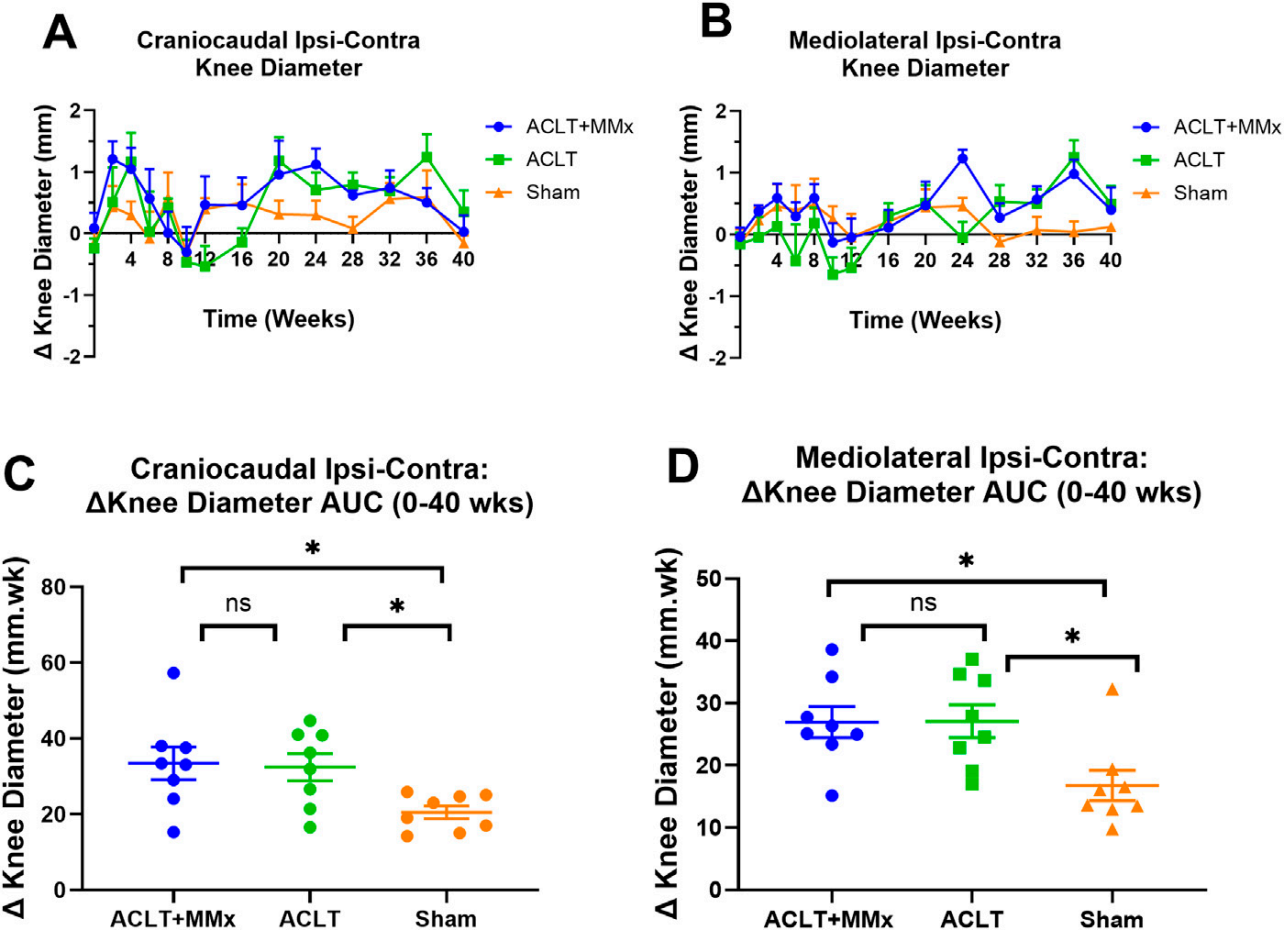
**(A)** Temporal changes in mean (±SEM) craniocaudal Δ knee diameter for each of the ACLT + MMx, ACLT and sham-groups of rats over a 40-week study period (n = 8/gp). **(B)** Mean (±SEM) mediolateral Δ knee diameter versus time curves for the ACLT + MMx, ACLT and sham-groups for the 40-week study period. **(C)** Mean (±SEM) area under the craniocaudal Δ knee diameter versus time curve values (Δ knee diameter AUCs) for the 0–40 weeks period differed significantly between the 3 groups (F_2,21_ = 4.550, P < 0.05) Dunnett’s multiple comparisons test showed that for both the ACLT + MMx and the ACLT groups, mean (±SEM) Δ knee diameter AUCs for the 0–40 weeks period differed significantly from the sham-group with the corresponding multiplicity adjusted P values of P < 0.05 and P < 0.05 respectively. **(D)** Mean (±SEM) area under the mediolateral Δ knee diameter versus time curve values (Δ knee diameter AUCs) for the 0–40 weeks period differed significantly between the 3 groups (F_2,21_ = 5.498 (P < 0.05) Dunnett’s multiple comparisons test showed that for both the ACLT + MMx and the ACLT groups, mean (±SEM) Δ knee diameter AUCs for the 0–40 weeks period differed significantly from the sham-group with the corresponding multiplicity adjusted P values of P < 0.05 and P < 0.05 respectively (One-way ANOVA with Dunnett’s multiple comparison test, *P < 0.05).

The mean (±SEM) pre-surgery craniocaudal diameters for the left and right knees for the various animal groups were as follows: ACLT + MMx: 11.9 (±0.3) and 11.8 (±0.3) mm respectively; ACLT: 11.3 (±0.3) and 11.6 (±0.4) mm respectively; Sham: 11.7 (±0.1) and 11.9 (±0.3) mm respectively and these values did not differ significantly (P > 0.05) between the knees and the groups (Figure 3A). Similarly, the mean (±SEM) pre-surgery mediolateral diameters for the left and right knees for the various animal groups were as follows: ACLT + MMx: 10.1 (±0.3) and 10.2 (±0.3) mm respectively; ACLT: 9.9 (±0.2) and 10.2 (±0.2) mm respectively; Sham: 10.1 (±0.1) and 10.2 (±0.2) mm respectively and these values did not differ significantly (P > 0.05) between the knees and the groups (Figure 3B). The mean (±SEM) baseline Δ knee diameters (ipsilateral–contralateral) for each of the craniocaudal and mediolateral dimensions prior to surgery did not differ significantly (P > 0.05) between rats in the ACLT + MMx, ACLT and sham-groups, consistent with expectations (Figures 3A, B). For the craniocaudal dimension, the mean (±SEM) Δ knee diameter values for the ACLT + MMx and ACLT groups peaked initially at week 4 and then decreased to baseline by 8–10 weeks post-surgery (Figure 3A). Interestingly, the mean (±SEM) craniocaudal Δ knee diameter values increased again at week 20 for the ACLT + MMx and ACLT groups before decreasing back to pre-surgery baseline values at week 40 (Figure 3A). For the mediolateral dimension, the mean (±SEM) Δ knee diameter values for the ACLT + MMx group peaked initially at week 4 and then decreased to baseline by ∼10 weeks post-surgery whereas for the ACLT group, the corresponding mean (±SEM) Δ knee diameter values did not differ significantly from the pre-surgery baseline values for the first 16-week of the model (Figure 3B). Subsequently, the mediolateral Δ knee diameter values increased at week 24 for the ACLT + MMx and at week 36 for both the ACLT + MMx and ACLT groups relative to the sham group (Figure 3B). Comparison of the craniocaudal Δ knee diameter AUC (0–40 weeks) values between the 3 groups (Figure 3C) showed that it differed significantly between the groups (F_2,21_ = 4.550 P < 0.05). Dunnett’s multiple comparisons test showed that for each of the ACLT + MMx and the ACLT groups, this parameter differed significantly from the sham-group with the corresponding multiplicity adjusted P values being P < 0.05 and P < 0.05 respectively. Similarly, the mediolateral Δ knee diameter AUC (0–40 weeks) values differed significantly between each of the ACLT + MMx and ACLT groups (F_2,21_ = 5.498, (P < 0.05) relative to the sham-group Figure 3D). Dunnett’s post hoc test showed that for both the ACLT + MMx and the ACLT groups, this parameter differed significantly from the sham-group with the corresponding multiplicity adjusted P values being P < 0.05 and P < 0.05 respectively.

### 3.3 Hindlimb static weight bearing asymmetry

**FIGURE 4:**
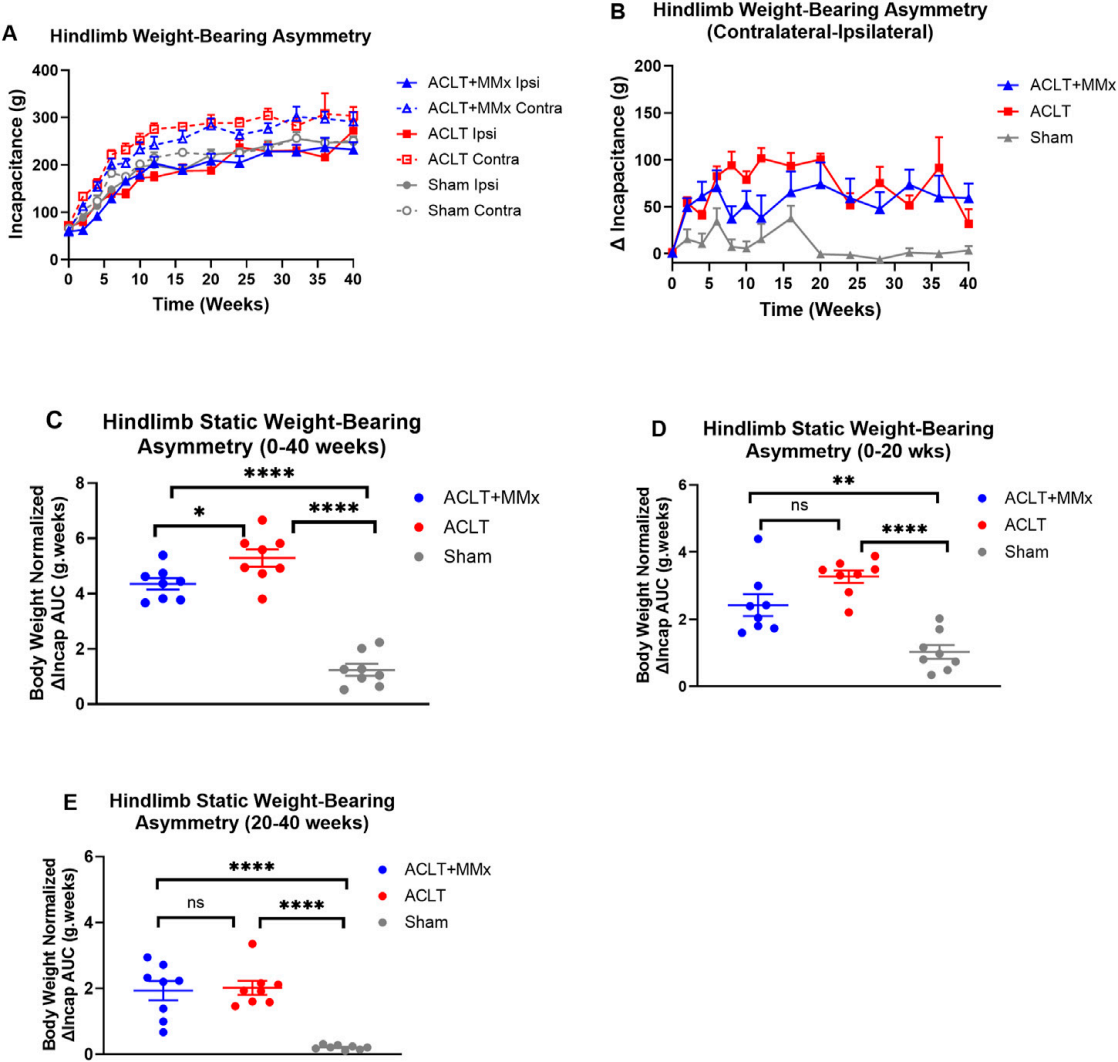
**(A)** Temporal changes in mean (±SEM) hindlimb incapacitance (g) for each of the ACLT + MMx, ACLT and sham-groups of rats over a 40-week study period (n = 8/gp). **(B)** Mean (±SEM) hindlimb static weight-bearing asymmetry between the contralateral and ipsilateral hindlimbs (ΔIncap) versus time curves for the ACLT + MMx, ACLT and sham-groups for the 40-week study period. **(C)** Mean (±SEM) body weight-normalized area under the ΔIncap versus time curve values (ΔIncap AUCs) for the 0–40 weeks period. The mean (±SEM) body weight normalized ΔIncap AUCs differed significantly (F_2,21_ = 73.44, P < 0.0001) between the three groups. Tukey’s post hoc test revealed that the body weight normalized ΔIncap AUCs differed significantly between the ACLT + MMx and the ACLT groups and the body weight normalized ΔIncap AUCs for each of the ACLT + MMx and the ACLT groups differed significantly from the corresponding value for the sham-group with the corresponding multiplicity adjusted P values being P < 0.05, P < 0.0001 and P < 0.0001 respectively. Mean (±SEM) body weight-normalized area under the ΔIncap versus time curve values (body weight-normalized ΔIncap AUCs) for the **(D)** 0–20 weeks period and for the **(E)** 20–40 weeks period. Additionally, for the 0–20 week interval, the body weight normalized ΔIncap AUCs differed significantly (F_2,21_ = 21.04, P < 0.0001) between the three groups. Tukey’s multiple comparisons test showed that the body weight normalized ΔIncap AUCs for each of the ACLT + MMx and ACLT groups differed significantly from that for the sham-group with the corresponding multiplicity adjusted P values being P < 0.01 and P < 0.0001 respectively. For the 20–40 week interval, the body weight normalized ΔIncap AUCs also differed significantly between the 3 groups (F_2,21_ = 24.13, P < 0.0001), a period when static weight-bearing asymmetry was minimal in the sham group. Tukey’s multiple comparisons test showed that the body weight normalized ΔIncap AUCs for each of the ACLT + MMx and ACLT groups for the 20–40 weeks interval differed significantly from the corresponding value for the sham-group with the corresponding multiplicity adjusted P values being P < 0.0001 and P < 0.0001 respectively. These findings show that static weight-bearing asymmetry is long-lasting and significant for distinguishing the ACLT + MMx and ACLT models from the sham-control group (One-way ANOVA with Tukey’s multiple comparison test, ****P < 0.0001; **P < 0.01; *P < 0.05; ns = not significant).

Prior to surgery, there were no significant differences (P > 0.05) in the static weight-bearing of either hindlimb for rats in the ACLT + MMx, ACLT and sham-groups or between the groups (Figure 4A). Specifically, the mean (±SEM) baseline incapacitance values for the left and right hindpaws for each of the three rat groups were: ACLT + MMx: 59.4 (±6.4) and 60.7 ± 6.6) g respectively; ACLT: 70.5 (±4.4) and 71.9 (5.5) g respectively; Sham: 63.6 (±2.5) and 66.2 (±2.6) g respectively (Figure 4A). Post-surgery, there were differential changes in static weight-bearing between the ipsilateral and contralateral hindlimbs for rats in the ACLT + MMx and ACLT groups relative to the sham-control group, that persisted for the 40-week study duration (Figure 4A). These changes are characterized by significant differences between the contralateral and ipsilateral static weight-bearing (ΔIncap) values for the ACLT + MMx and ACLT-groups relative to the sham-group (Figures 4B–E), and particularly for the 20–40 week period post-model induction upon visual inspection (Figures 4B, E). Specifically, the mean (±SEM) area under the body weight-normalized ΔIncap versus time curve values (body weight normalized ΔIncap AUCs) differed significantly (F_2,21_ = 73.44, P < 0.0001) between the three rat groups. Tukey’s post hoc test revealed that the body weight normalized ΔIncap AUCs differed significantly between the ACLT + MMx and the ACLT groups (P < 0.05) and for both these groups, the body weight normalized ΔIncap AUCs differed significantly from the corresponding value for the sham-group with the corresponding multiplicity adjusted P values being P < 0.0001 and P < 0.0001 respectively (Figure 4C). Additionally, for the 0–20 week interval (Figure 4D) the body weight normalized ΔIncap AUCs differed significantly (F_2,21_ = 21.04, P < 0.0001) between the three groups. Tukey’s multiple comparisons test revealed that the body weight normalized ΔIncap AUCs for each of the ACLT + MMx and ACLT groups differed significantly from the corresponding value for the sham-group with the corresponding multiplicity adjusted P values being P < 0.01 and P < 0.0001 respectively (Figure 4D). For the 20–40 week interval, the body weight normalized ΔIncap AUCs also differed significantly between the 3 groups (F_2,21_ = 24.13, P < 0.0001) (Figure 4E), a period when static weight-bearing asymmetry was minimal in the sham group. Tukey’s multiple comparisons test revealed that the body weight normalized ΔIncap AUCs for each of the ACLT + MMx and ACLT groups for the 20–40 week interval differed significantly from the corresponding value for the sham-group with the corresponding multiplicity adjusted P values being P < 0.0001 and P < 0.0001 respectively (Figure 4E). These findings together show that hindlimb static weight-bearing asymmetry is a robust and significant pain behavioral parameter that persists over a prolonged study period (40-week) and so has potential for use in studies designed to assess novel OA disease-modifying therapeutics over several months as well as novel investigational analgesic agents for the relief of OA knee pain.

### 3.4 Mechanical allodynia in the hindpaws

**FIGURE 5:**
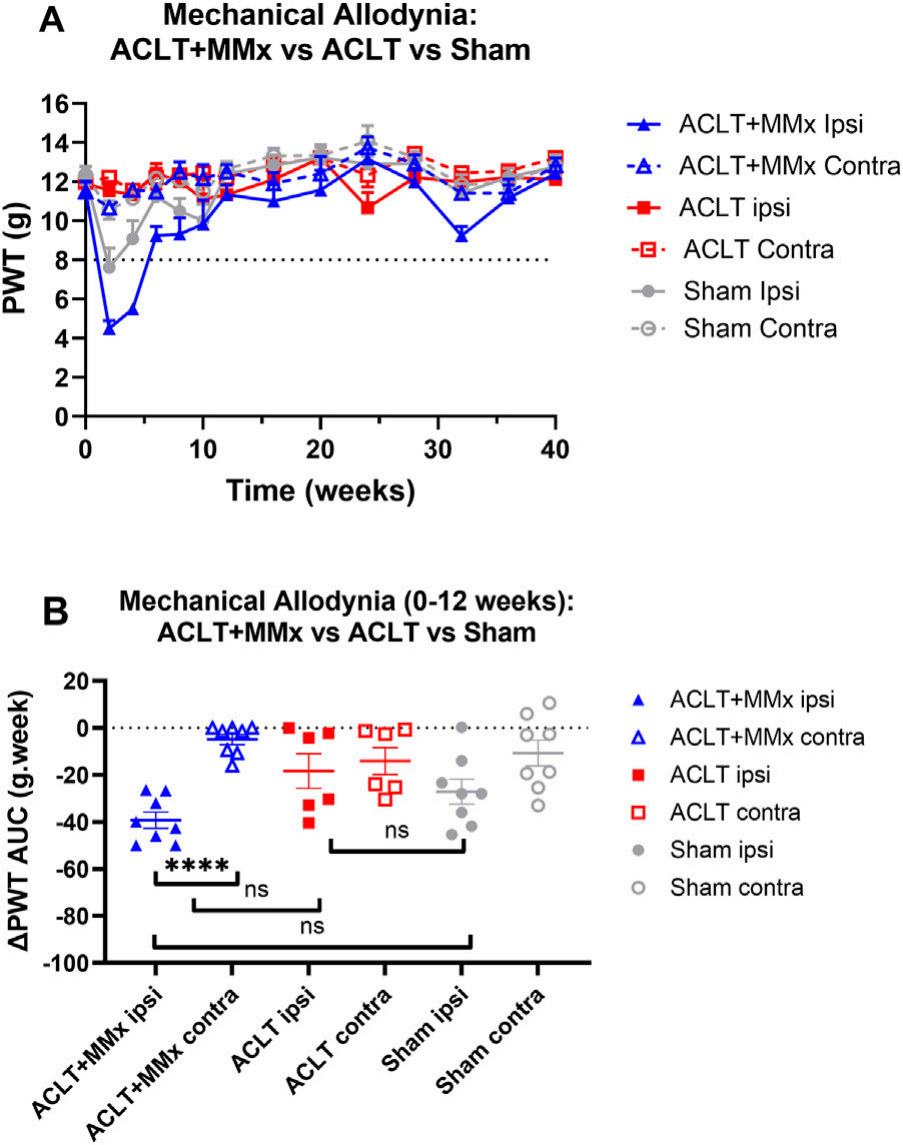
Mechanical allodynia was fully developed in the ipsilateral (but not the contralateral) hindpaws of the ACLT + MMx rat model of OA knee pain (PWTs ≤ 8 g) at 2 and 4-weeks post-model induction. **(A)** Mean (±SEM) PWT versus time curves for the ipsilateral and contralateral hindpaws for the ACLT + MMx, ACLT and sham-groups of rats for the 40-week study period. **(B)** The mean (±SEM) extent and duration (AUC) of the decrease in the ipsilateral PWTs relative to pre-surgery baseline PWTs (ΔPWT AUC values) differed significantly (F_5,38_ = 6.788, p < 0.0001) between the 3 groups. Tukey’s multiple comparisons test revealed that for the ACLT + MMx group the mean (±SEM) ipsilateral ΔPWT AUC value for the period 0–12 weeks post-model induction differed significantly from that for the contralateral hindpaws of the same group but not the ipsilateral hindpaws of the ACLT or the sham-groups with the corresponding multiplicity adjusted P values being P < 0.0001, P > 0.05 and P > 0.05 respectively. Similarly, Tukey’s multiple comparisons test showed that the ΔPWT AUC values for the ipsilateral hindpaws of the ACLT-group did not differ significantly from the corresponding values for the sham group (P > 0.05) (One-way ANOVA with Tukey’s multiple comparison test. ****P < 0.0001; ns = not significant).

Prior to surgery, the baseline PWTs did not differ between the left and right hindlimbs or amongst the 3 rat groups (P > 0.05). Specifically, the mean (±SEM) baseline PWT values for the left and right hindpaws for each of the 3 rat groups were: ACLT + MMx: 11.5 (±0.5) and 11.5 ± 0.5) g respectively; ACLT: 11.9 (±0.2) and 12.0 (0.2) g respectively; Sham: 12.2 (±0.4) and 12.3 (±0.4) g respectively (Figure 5A). Post-surgery, there was temporal development of mechanical allodynia in the ipsilateral hindpaws of rats in the ACLT + MMx group that was fully developed (PWTs ≤8 g) at 2- and 4-week post-model induction (Figure 5A). Thereafter, visual inspection shows that the ipsilateral PWTs returned to baseline levels by ∼8–12-weeks which were maintained until study completion at 40-week (Figure 5A). Mechanical allodynia did not develop in the contralateral hindpaws of the ACLT + MMx group or in either hindpaw of the ACLT group (Figure 5A). For the sham-group, fully developed mechanical allodynia (PWTs ≤8 g) was observed in the ipsilateral hindpaws at 2-weeks post-surgery only and mechanical allodynia did not develop in the contralateral hindpaws of the same animals (Figure 5A). For the hindpaws, the mean (± SEM) extent and duration (AUC) of mechanical allodynia (ΔPWT AUC values) differed significantly (F_5,38_ = 6.788, p < 0.0001) between the 3 groups. Tukey’s multiple comparisons test showed that for the ACLT + MMx group, the mean (±SEM) ipsilateral ΔPWT AUC value for the period 0–12 weeks post-model induction differed significantly from that for the contralateral hindpaws of the same group but not the ipsilateral hindpaws of the ACLT or the sham-groups with the corresponding multiplicity adjusted P values being P < 0.0001, P > 0.05 and P > 0.05 respectively (Figure 5B). Similarly, Tukey’s multiple comparisons test showed that the ΔPWT AUC values for the ipsilateral hindpaws of the ACLT-group did not differ significantly from the corresponding values for the sham group (P > 0.05) (Figure 5B).

### 3.5 Mechanical hyperalgesia in the hindpaws

**FIGURE 6:**
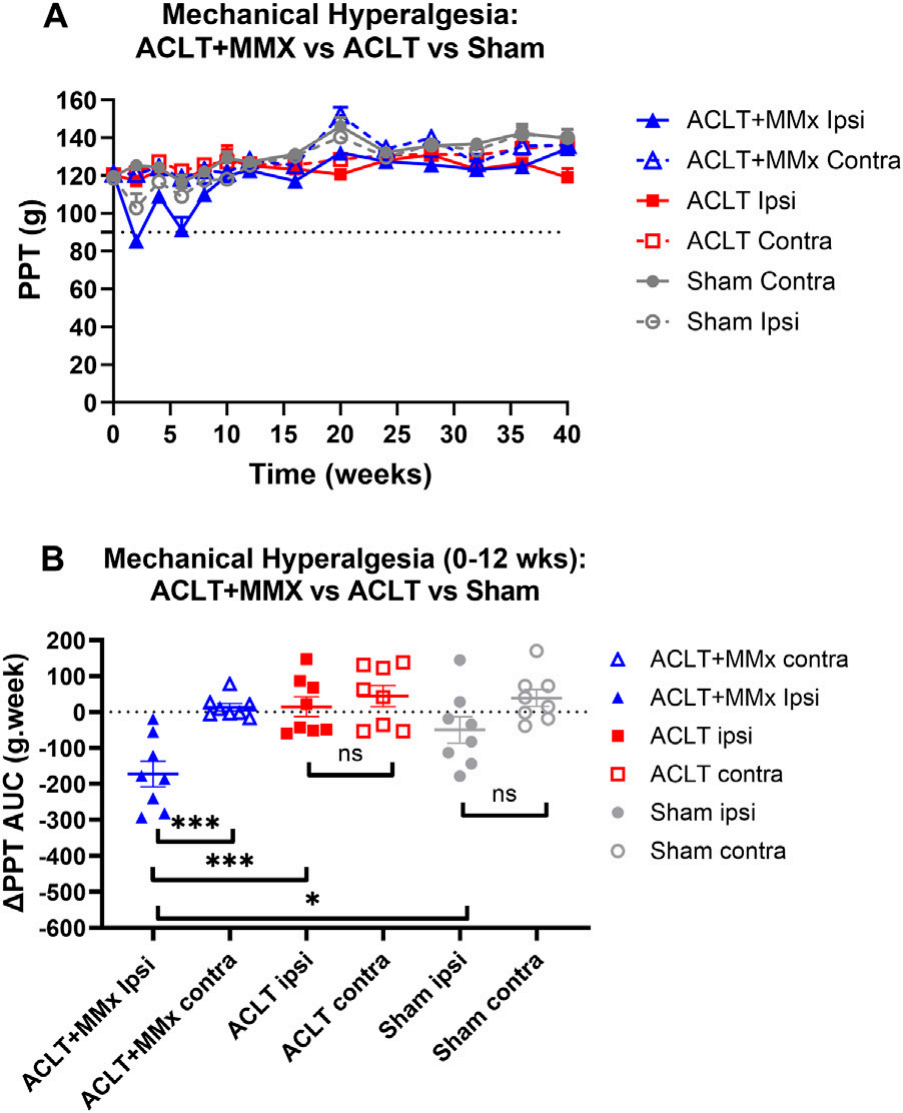
Mechanical hyperalgesia was fully developed in the ipsilateral (but not the contralateral) hindpaws (PPTs ≤90 g) of the ACLT + MMx rat model at 2–6 weeks post-model induction. **(A)** Mean (±SEM) PPT versus time curves for the ipsilateral and contralateral hindpaws of ACLT + MMx, ACLT and sham-groups of rats for the 40-week study period. **(B)** For the ACLT + MMx group, the mean (±SEM) extent and duration (area under the curve) of mechanical hyperalgesia that developed in the ipsilateral hindpaws for the 0–12 weeks period post-model induction (ΔPPT AUC values) differed significantly (F_5,42_ = 8.298, p < 0.001) between the 3 groups. Tukey’s multiple comparisons test revealed that for the ACLT + MMx group, the mean (±SEM) ΔPPT AUC values for the first 12-weeks post-surgery differed significantly from the corresponding values for the contralateral hindpaws of the same animals as well as from the ΔPPT AUC values for both hindpaws of the ACLT and sham-groups with the corresponding multiplicity adjusted P values being (P < 0.001, P < 0.001, P < 0.05 respectively. For the ACLT-group, Tukey’s multiple comparisons test showed that the mean (±SEM) ΔPPT AUC values did not differ significantly (P > 0.05) between the hindpaws. Similarly for the sham group, Tukey’s multiple comparisons test showed that the mean (±SEM) ΔPPT AUC values did not differ significantly (P > 0.05) between the ipsilateral and contralateral hindpaws, consistent with expectations (One-way ANOVA with Tukey’s multiple comparison test, ***P < 0.001; *P < 0.05; ns = not significant).

Prior to surgery, the baseline PPTs did not differ between the left and right hindlimbs or amongst the 3 rat groups (P > 0.05). Specifically, the mean (±SEM) baseline PPT values for the left and right hindpaws for each of the 3 rat groups were: ACLT + MMx: 120.6 (±1.6) and 121.0 ± 1.4) g respectively; ACLT: 120.4 (±2.5) and 120.4 (2.9) g respectively; Sham: 118.5 (±1.9) and 119.8 (±2.0) g respectively (Figure 6A). Post-surgery for the ACLT + MMx group of rats, mechanical hyperalgesia was fully developed (PPTs ≤90 g) in the ipsilateral (but not the contralateral) hindpaws at 2- and 6-weeks post-model induction (Figure 6A). Visual inspection shows that mean (±SEM) ipsilateral PPTs returned to pre-surgery baseline values by ∼8–12 weeks and remained at baseline levels until study completion at 40-weeks (Figure 6A). For the hindpaws, the mean (±SEM) extent and duration (AUC) of mechanical hyperalgesia (ΔPPT AUC values) differed significantly (F_5,42_ = 8.298, p < 0.001) between the 3 groups. Tukey’s multiple comparisons test revealed that for the ACLT + MMx group, the mean (±SEM) ΔPPT AUC values for the first 12-week post-surgery differed significantly from the corresponding mean (±SEM) ΔPPT AUC value for the contralateral hindpaws of the same animals as well as from the corresponding ΔPPT AUC values for the ipsilateral hindpaws of the ACLT- and the sham-groups with the corresponding multiplicity adjusted P values being (P < 0.001, P < 0.001, P < 0.05 respectively (Figure 6B). For the ACLT-group, mechanical hyperalgesia did not develop in either the ipsilateral or the contralateral hindpaws (Figure 6A) and Tukey’s multiple comparisons test showed that the associated mean (±SEM) ΔPPT AUC values did not differ significantly (P > 0.05) between the hindpaws (Figure 6B). Similarly for the sham group, mechanical hyperalgesia did not develop in either the ipsilateral or the contralateral hindpaws (Figure 6A) and Tukey’s multiple comparisons test showed that the mean (±SEM) ΔPPT AUC values did not differ significantly between the ipsilateral and contralateral hindpaws, consistent with expectations (Figure 6B).

## 4 Discussion

Herein, we show long-lasting (40-weeks), significant hindlimb static weight-bearing asymmetry in the ACLT + MMx and the ACLT rat models of OA knee pain. Our findings extend previous work on hindlimb static weight-bearing asymmetry in the ACLT + MMx rat model of knee OA in two 12-week studies (Kao et al., 2016; Luo et al., 2021) and one 8-week study (Hamilton et al., 2015). The long-lasting and significant hindlimb static weight-bearing asymmetry suggests that this pain behavioral endpoint has potential utility for assessing the efficacy of novel OA disease-modifying agents and/or well-tolerated pain therapeutics for relief of chronic unrelenting OA knee pain in patients. The relevance of our findings is high as the heavy reliance to date on modulation of histologic changes in rodent models of OA knee pain with scant attention to pain behavioral endpoints, has failed to deliver novel disease modifying agents and/or well-tolerated analgesics for alleviating unrelenting OA knee pain.

Our findings showing long-lasting hindlimb static weight-bearing asymmetry in the ACLT + MMx and ACLT rat models of OA knee pain, are aligned with histopathological changes reported by others in a 20-week study using the ACLT + MMx rat model. Specifically, histopathology and micro-CT imaging showed ipsilateral joint surface discontinuity by 2-weeks post-model induction that persisted at 4-weeks, along with early proteoglycan loss but the latter was not progressive (Appleton et al., 2007). Also, there was a temporal increase in the OARSI (Osteoarthritis Research Society International) histopathology grading and staging scores for both surfaces of the ipsilateral knee joint, with maximal scores observed at 8-weeks that persisted until study completion at 20-week (Appleton et al., 2007). Importantly, the OARSI scores for the ipsilateral joints of the sham-animals, did not differ significantly from zero and the corresponding micro-CT images showed no evidence of damage in the ipsilateral knees (Appleton et al., 2007). Nevertheless, the lack of correlation between histopathologic features of OA and symptomatic presentation is a long-standing challenge in the field of osteoarthritis pain^5^.

In other work that used a unilateral meniscal transection model of knee OA in young male SD rats, significant hindlimb weight-bearing asymmetry was evident by 4-weeks and it persisted until study completion at 7-weeks (Mapp et al., 2013). Similarly, there was hindlimb weight-bearing asymmetry evident by 1-week after unilateral ACLT in young male Wistar rats that persisted until study completion at 8-weeks (Tawonsawatruk et al., 2018). Although a short term (3–4 weeks) study in the milder medial meniscus transection (MMT) model in the young male Lewis rat model of OA knee pain, reported no significant differences in hindlimb static weight-bearing relative to the sham-group (Allen et al., 2012), more recent work showed significant reproducible changes in hindlimb jump incapacitance in the mildly destabilized medial meniscus (DMM) rat model of OA of the knee over a 90-day study period (Westhof et al., 2021).

In work that used male, skeletally mature, Lewis rats at 3 months of age to surgically induce a unilateral ACLT + pMMx OA knee model, significant hindlimb static weight-bearing asymmetry was observed at one and at 5-weeks post-surgery, but it resolved spontaneously by 10-weeks post-model induction despite the persistent histopathological changes in the operated knee at 10-weeks (Ripmeester et al., 2024). In a similar study in male Lewis rats at 12-weeks of age and given a unilateral ACLT + pMMx surgical procedure to induce knee OA, dynamic weight-bearing asymmetry was evident by 1-week and it persisted until 4-weeks post-surgery (Henrotin et al., 2021). Thereafter, dynamic weight-bearing asymmetry resolved spontaneously such that the weight bearing of the operated hindlimb increased significantly by 27% at week 5 and by 37% by week 8 (Henrotin et al., 2021). Possible explanations for the short-lived (4–5 weeks) nature of the weight-bearing asymmetry observed in these latter two studies in contrast to the long-lasting (40-weeks) hindlimb static weight-bearing asymmetry in the ACLT + MMx and ACLT rat models of OA knee pain herein, is that there were rat strain differences (Lewis versus Sprague-Dawley) and this requires future investigation.

In previous work using the DMM rat model of knee OA, there was persistent joint broadening (increased mediolateral Δ knee diameter) from 1-week that persisted until study completion at 13-weeks (Westhof et al., 2021). Herein, the mediolateral Δ knee diameter was minimal for the ACLT group for the first 24-weeks but it did increase thereafter peaking at week 36 suggestive of joint instability. For the ACLT + MMx group, the mediolateral Δ knee diameter increased relative to the baseline for ∼8-weeks. From ∼ week 20 in these animals, the mediolateral Δ knee diameter increased in a second phase which peaked at week 36, again suggestive of joint instability. Interestingly, for the craniocaudal dimension, there was an initial phase of ipsilateral knee swelling for both the ACLT + MMx and ACLT rat groups characterized by an increase in the Δ knee diameter that resolved by ∼8–10 weeks post-model induction. This was followed by a second phase of joint swelling (increase in the craniocaudal Δ knee diameter) from ∼20-week, aligned with the possibility of joint instability, that decreased to baseline values at 40-weeks. Factors potentially contributing to the development of joint instability include inflammatory processes, cartilage degradation and biomechanical shifts (Tong et al., 2022). Overall, however, these Δ knee diameter changes were less marked than the magnitude and persistence of the hindlimb static weight bearing asymmetry between the two OA models and that of the control group.

Other pain behavioral endpoints evaluated in surgically induced rodent OA knee models include mechanical allodynia and/or mechanical hyperalgesia in the hindpaws (Allen et al., 2012; Miotla Zarebska et al., 2017). In work where rats underwent a unilateral ACLT + pMMx procedure, mechanical hyperalgesia was evident in the ipsilateral hindpaws at 1-week post-surgery but it was reduced to low intensity by week 2 which was maintained until study completion at week 8 (Henrotin et al., 2021). This spontaneous reduction in mechanical hypersensitivity in the ipsilateral hindpaws is similar to our findings herein whereby the mechanical hypersensitivity (allodynia and hyperalgesia) that developed in the ipsilateral hindpaws of the ACLT + MMx rat model of OA knee pain resolved spontaneously by ∼8–12 weeks post-model induction. Comparison of the extent and duration of the decrease in ipsilateral PWTs and PPTs relative to baseline values (ΔPWT AUCs and ΔPPT AUCs respectively) for the ACLT + MMx group relative to the ACLT and the sham-groups herein, showed significant differences for the 0–12 weeks period post-model induction (Figures 5, 6) but not between the ACLT and sham-groups. Neither mechanical allodynia nor mechanical hyperalgesia developed in the contralateral hindpaws of any of the animals in the present study. The significant mechanical allodynia for the 0–12 weeks period after ACLT + MMx surgery in rats herein, is aligned with that of a short-term study (3–4 weeks) in the milder MMT-rat model of OA knee pain whereby mechanical allodynia developed in the ipsilateral but not the contralateral hindpaws between 9- and 24-days post-model induction (Allen et al., 2012).

Mechanisms potentially contributing to the development of OA knee pain include sensitization of primary sensory neurons, neuroinflammation and structural changes in the diseased joint including increased sensory innervation and development of synovial fibrosis, central sensitization, reduced descending inhibition and atrophy of cortical areas (Eitner et al., 2017; Miller et al., 2020; Palada et al., 2020; Zhang et al., 2022). Pronociceptive mediators implicated in these mechanisms include classical inflammatory mediators (e.g., prostaglandin E2, bradykinin, histamine, protons, and 5-hydroxytryptamine), neuropeptides (e.g., calcitonin gene related peptide, substance P), adrenergic mediators, pro-inflammatory cytokines, neurotrophins, damage-associated molecular patterns, pathogen-associated molecular patterns, signalling via their cognate receptors and/or ion channels (Eitner et al., 2017; Aso et al., 2020). At present, it is unclear if convergent mechanisms mediate the pathobiology of OA disease and OA pain or whether these need to be treated as separate entities for novel therapeutics discovery (Eitner et al., 2017). Future research is required to investigate the contributions of these various factors including possible temporal changes in the concentrations of various pro- and anti-inflammatory cytokines and the extent to which these changes contribute to the pathobiology of persistent hindpaw static weight-bearing asymmetry in the MMx + ACLT and the ACLT rat models of OA knee pain.

A limitation of the present exploratory study is that one rat sex (male) was used, and this will be addressed in our planned future work that will use both sexes. Although another limitation is that temporal changes in histological endpoints were not assessed, there are numerous studies that have documented histological changes in rat models of OA in the knee joint of the models used herein. Now that we have identified hindlimb static weight bearing asymmetry as a significant pain behavioral endpoint that persists for at least 40-weeks after induction of the ACLT + MMx and ACLT rat models of OA knee pain, our future work will also assess temporal changes in knee histology in parallel with temporal changes in hindlimb static weight bearing asymmetry over a 40-week period post-model induction. Our future work will also assess the impact of pharmacological agents on these endpoints.

## 5 Conclusion

Unrelenting OA knee pain is a primary reason that patients seek medical treatment or progress to having knee replacement surgery (Malfait and Schnitzer, 2013). The unmet need for novel, well-tolerated and highly effective analgesic agents for the relief of chronic OA knee pain requires that methods for assessing pain behavior over prolonged periods in rodent OA knee pain models, are needed. Herein, we showed that for both the widely utilized and standardized ACLT + MMx and ACLT rat models of surgically induced OA knee pain, hindlimb static weight-bearing asymmetry is a long-lasting, reproducible and highly significant pain behavioral parameter, particularly in the advanced stages of these models in the period 20–40 weeks post-model induction. By contrast, mechanical hypersensitivity in the ipsilateral hindpaws of the ACLT + MMx rat model resolved spontaneously by 8–12 weeks and mechanical hypersensitivity did not develop in the hindpaws of the ACLT-group like the sham-group. Thus, hindlimb static weight-bearing asymmetry has utility for assessing novel disease-modifying OA therapeutics and/or well-tolerated analgesic drug candidates aimed at alleviating unrelenting chronic OA knee pain in patients.

## Data Availability

The raw data supporting the conclusions of this article will be made available by the authors, without undue reservation.
